# The development of FEDUPP: feeding experimentation device users processing package to assess learning and cognitive flexibility

**DOI:** 10.1038/s41398-026-04091-6

**Published:** 2026-05-16

**Authors:** Mingyang Yao, Avraham M. Libster, Shane Desfor, Freiya Malhotra, Nathalia Castorena, Patricia Montilla-Perez, Francesca Telese

**Affiliations:** 1 Department of Mathematics, School of Physical Sciences, University of California, San Diego, CA USA; 2 Department of Psychiatry, School of Medicine, University of California, San Diego, CA USA; 3 School of Biological Sciences, University of California, San Diego, CA USA

**Keywords:** Neuroscience, Psychology

## Abstract

Cognitive flexibility, the ability to adapt behavior in response to changing contingencies, is a key component of adaptive decision-making and is impaired in multiple neuropsychiatric disorders. Traditional rodent assays of cognitive flexibility are conducted in experimenter-controlled sessions in restrictive environments, limiting ecological validity and temporal resolution. Here, we developed a fully automated, home-cage paradigm using the Feeding Experimentation Device 3 (FED3) and a companion open-source analysis pipeline, the Feeding Experimentation Device Users Processing Package (FEDUPP), to assess learning and cognitive flexibility with minimal experimenter intervention. The paradigm combines a single-day fixed-ratio 1 (FR1) task with a multi-day, reversal learning task in which active port assignment switches every 25 pellets collected. FEDUPP implements multi-scale learning metrics, including overall accuracy, an 80% accuracy milestone, and a machine learning-based classification of meal accuracy to capture motivated, goal-directed feeding. In wild-type mice, the paradigm detected rapid FR1 acquisition and progressive within-block adaptation during reversal. Application to mice with dorsal hippocampal knockdown of the scaffolding protein CASK revealed faster FR1 acquisition and higher accuracy. In addition, a faster onset of the first accurate meal after reversal suggests an improvement in updating goal-directed feeding behavior. These findings demonstrate that FEDUPP enables high-resolution, continuous assessment of learning and cognitive flexibility in ethologically relevant settings, and that meal-based accuracy provides a sensitive metric for detecting subtle changes in flexibility not captured by traditional measures.

## Introduction

Cognitive flexibility is the ability to adapt behaviors and thought processes in response to changing environments, enabling individuals to revise strategies, switch tasks, and adopt new response patterns when reward structures or task demands shift [1–3]. This adaptability is necessary when suppressing previously learned behaviors to achieve new goals [4].

Underlying cognitive flexibility is a network of neural processes that enable the efficient and dynamic allocation of cognitive resources [5]. While much of the focus has traditionally been on the prefrontal cortex and basal ganglia [5–8], the hippocampus also plays a significant role due to its involvement in contextual processing [9]. Deficiencies in cognitive flexibility contribute to maladaptive behaviors seen in disorders such as schizophrenia, obsessive-compulsive disorder, and addiction, highlighting the importance of understanding their underlying neural mechanisms [10–12].

Numerous behavioral assays have been developed to investigate the neural basis of cognitive flexibility in both human and animal models. These methods are based on reversal learning paradigms and involve adapting behavior when previously rewarded cues are reversed [13, 14]. These include the Wisconsin Card Sorting Test (WCST) [15, 16], T-maze and Y-maze tasks [17, 18], instrumental discrimination reversal tasks [19–21], and operant chamber tasks in contexts of probabilistic reward shifts [22, 23]. These methods have demonstrated the important roles of the prefrontal cortex and nucleus accumbens in regulating flexible behavior [20, 24]. Despite the valuable insights gained from these methods, they come with limitations.

Behavioral assays have been conducted within experimenter-controlled sessions in restrictive environments, which can affect natural learning and behavior due to factors such as handling-related stress and the novelty of unfamiliar environments. Moreover, traditional behavioral analyses in cognitive flexibility research often rely on summary metrics such as cumulative accuracy or error rates, which do not fully capture the dynamics of learning and adaptation over time [2]. These metrics often fail to represent the continuous process of behavioral adjustment and may overlook critical transitions between different states, such as exploration, where new strategies are tested to gather information, and exploitation, where known successful behaviors are used to maximize rewards [1]. Finally, manual video scoring methods introduce potential inaccuracies due to human error and subjectivity, limiting the resolution of complex behaviors [25]. This limitation underscores the need for more sophisticated methods to analyze nuanced behavioral adaptations, which can reflect complex cognitive processes like reinforcement learning involving trial-and-error learning to optimize actions and flexible decision-making that balances exploration and exploitation.

To address these challenges, the Feeding Experimentation Device 3 (FED3) was utilized in a home-cage environment, enabling continuous, minimally invasive tracking of operant learning and behavioral adaptation [26, 27]. In addition, a behavioral assay protocol and accompanying software toolbox, FEDUPP (Feeding Experimentation Device Users Processing Package), was developed to assess learning and cognitive flexibility. This allowed animals to autonomously engage with reversal tasks in their home cage, preserving naturalistic behaviors across circadian cycles and reducing stress-related confounds. Furthermore, new metrics to evaluate learning efficiency and transitions were established. To that end, machine learning for detailed behavior classification was used.

Following the establishment of the assay and analysis framework, they were validated under conditions of known neural disruption. For this, male C57BL/6 mice underwent targeted knockdown (KD) of CASK (Calcium/Calmodulin-Dependent Serine Protein Kinase) in the dorsal hippocampus via shRNA-mediated gene silencing (CASK-KD). Littermate controls received hippocampal injections of a non-targeting shRNA construct (Control). This perturbation model enabled the assessment of the sensitivity of behavioral assays and analytical metrics in detecting cognitive flexibility changes resulting from molecular disruption.

These innovations provide a refined and scalable approach for investigating cognitive flexibility and adaptive learning in animal models, capturing the complexity of learning processes crucial for informed decision-making.

## Methods

### Ethics declarations

All experimental procedures were conducted in accordance with the guidelines established by the National Institutes of Health Guide for the Care and Use of Laboratory Animals and approved by the Institutional Animal Care and Use Committee (IACUC) at the University of California, San Diego.

### Mice

Male and female mice were housed (2–5 per cage), aged 10–20 weeks, under a 12 h light/12 h dark cycle and provided with food and water ad libitum. At the start of the behavioral paradigms, mice were single-housed. Experiments were conducted using age-matched C57BL/6J (Strain #:000664, JAX) mice in each cohort. 20 female mice were used for method development, and 66 male mice for the CASK-KD experiment. Two mice were removed from the FR1 and 2 from the reversal phases of the WT group due to unrecoverable timestamp errors or excessively long blocking periods when the FED3 device dispenses a pellet. Therefore, we included 18 mice in FR1 and 18 mice in the reversal phase. For comparison analysis, in the FR1 experiment, the CASK-KD condition and Control had 31 and 35 mice, respectively. In the reversal task, the CASK-KD condition and Control had 24 and 27 mice, respectively. The reduced number of mice between the tasks is due to a cohort of mice that underwent only the FR1 task. In addition, due to a technical issue, one mouse in the CASK-KD had less than 2 days of data and was removed from the analysis.

### Stereotaxic surgeries

Mice were anesthetized using 5% isoflurane and then placed on a stereotaxic frame (Model 68803, RWD, Shenzhen, China). An incision was made to expose the skull, and the skull was leveled relative to bregma and lambda. Bilateral craniotomies were performed using a drill. Viral preparations (AAVDJ-CMV-LACz-U6-shCASK or AAVDJ-CMV-eGFP-U6-shCASK for the CASK-KD group, and AAVDJ-CMV-LACZ-U6-shScramble or AAVDJ-CMV-eGFP-U6-shScramble for the Control group, with a titer of ~4–5 × 10^12) were injected through a glass pipette using a Nanoject III (Drummond Scientific, Broomall, USA). Coordinates of injections were (AP:−2 ML:0.9 DV: −2.15, −1.65, AP:−2 ML:1.4 DV: −2.1, −1.35, AP:−2 ML:2 DV: −2.1,−1.45) with injection volume of 120–200 nl in each site at a rate of 3–5 nl/sec. Following surgery, the wound was stitched, and the mouse was given a subcutaneous injection of ethiqaXR (3 mg/kg). Mice were allowed to recover for at least 3 weeks before the start of the experiments.

### AAV constructs

AAV plasmid constructs were designed and cloned by VectorBuilder, and their maps and sequences can be retrieved using the vector IDs at https://vectorbuilder.com. Scramble shRNA AAV vector IDs are VB180117-1020znr and VB230831-1460avd, and shRNA-CASK AAV vector IDs are VB200205-1157jmg and VB230831-1459ard. AVV vectors included either an EGFP or LacZ reporter gene. Vectors were packaged into a virus by the Gene Transfer, Targeting, and Therapeutics Viral Vector Core at the Salk Institute for Biological Studies.

### shRNA-mediated CASK-KD validation by RT-qPCR

Mouse neuroblastoma N2a (CCL-131, ATCC) cells were plated in 24-well plates and transfected with AAV plasmids encoding either shCask or scramble shRNA control (both vectors carried an EGFP reporter) using LipoD293 (#SL100668, SignaGen Laboratories) reagent according to the manufacturer’s protocol. Cells were harvested 48 h after transfection. Total RNA was extracted using the Direct-zol RNA Kit (#R2050, Zymo Research), treated with DNase I, and reverse transcribed using the Maxima H Minus First Strand cDNA Synthesis Kit (#K1652, ThermoFisher Scientific). qPCR was performed with SsoAdvanced Universal SYBR® Green Supermix (#1725270, Biorad) using primers specific for *Cask* and the housekeeping gene *Actb*, as follows: Cask-Forward = ATGGGGGTATGATTCACAGG, Cask-Reverse = CTGATGCCATTGATTTCTCG, Actb-Forward = CCTGGATGGCTACGTACATGGCTG, Actb-Reverse = ACCTTCTACAATGAGCTGCGTGTG. Each biological replicate (independent transfection) was run in technical replicates, and technical replicates were averaged. For normalization, Ct were converted to DeltaCt = Ct(Cask) - Ct(Actb). Relative expression was calculated as 2^-DDCt = mean DC(sample) - mean DCt(scramble). Data are plotted as mean +/− standard deviation of biological replicates. Statistical comparisons between shScramble and shCASK groups used an unpaired t-test.

### IHC for viral infection validation

Mice were anesthetized using IP injection of Pentobarbital (50 mg/Kg) and perfused using 4% PFA. Brains were kept overnight in 4% PFA and then transferred to 30% sucrose solution until they sank. Following the brain sinking, they were coronally sliced (16 μm thickness) using a sliding vibratome (Microm HM 440E, MicroM, Germany) and kept in a cryoprotectant solution (Hoffman, Murphy and Sita, 2016). Slices containing hippocampus were mounted on glass slides and then blocked with PBST (PBS + 0.3% TritonX) + 5% NGS. Following blocking, slices were exposed to anti-GFP primary antibody (GFP 1020, Aves Labs, Davis, California, USA) at a dilution of 1:1000 overnight at 4 °C. Then, they were washed in PBST and exposed to anti-chicken 488 secondary antibody (103-545-155, Jackson Immunoresearch, Westgroove, PA, USA) for one hour at room temperature. Then, they were washed and stained with DAPI (1.2 μM, Sigma Aldrich) for 30 min. Following DAPI slides were washed and then coverslipped using mounting media (DAVCO, Sigma Aldrich). Slices were imaged using a Keyence BZ-X800 (Keyence, Itasca, IL, USA.).

### FR1 and reversal behavioral protocols

Operant learning was evaluated using the FED3, which allows mice to be trained to nose-poke for food pellets with minimal experimenter involvement. FED3 devices were purchased from Open-Ephys (https://open-ephys.org/fed3*)*. FED3 tasks were conducted with the mice housed individually, with water available ad libitum. Food was accessible exclusively through the FED3, delivering 20 mg pellets (20 mg 5TUM pellets (1811143), TestDiet). Each nose-poke, in either port, and pellet retrieval event was automatically recorded by each FED3, with data saved in CSV format on an SD card. Detailed analyses of FED tasks for specific cohorts are provided below. FR1 paradigm was realized using code supplied in the FED3 open source library, and the reversal paradigm was a modified version of the ProbReversalTask.ino from the FED3 library (https://github.com/KravitzLabDevices/FED3_library). The modified script is available in https://github.com/ftlabucsd/FEDUPP under scripts/ClassicFED3_WithReversalTask.ino.

#### Fixed ratio 1 (FR1)

During the FR1 task, one of the two nose ports was designated as ‘active’. A single poke in that port resulted in a pellet being dispensed. The other port was ‘inactive’, whereas a nose poke did not result in a pellet being dispensed. Mice were engaged with the FR1 task for 24–30 h.

#### Reversal learning

The reversal learning task was composed of consecutive blocks. During each block, the mouse performed an FR1 task, based on a specific port, either right or left, being the active port. Once 25 pellets were dispensed, the block was switched to the next block. In the consecutive block, the mouse performed an FR1 task, but now the roles of the ports, either active or inactive, were reversed. The reversal learning task started immediately after the FR1 task.

### Analysis programming tools

For structuring, processing, and analyzing data generated from experiments conducted with the FED3, we utilized Python (version 3.9) as the primary programming language. Key libraries included pandas, numpy, and datetime, which were employed for data manipulation and temporal handling, while matplotlib and seaborn were used for data visualization. Code is available at https://github.com/ftlabucsd/FEDUPP.

### Metrics used in the FR1 and reversal task

The following are definitions and custom metrics developed based on both prior research and specific customizations for the behavioral dataset.

#### Meal definition

A meal began when two pellets were retrieved within 60 s of each other, and additional pellets were included as long as each subsequent retrieval occurred within 60 s of the first pellet retrieval. Once a retrieval event exceeded the 60-second threshold, the meal was considered complete, and no additional pellets were included. Finally, the sequence of pellet retrieval was considered to be a meal only if the proportion of pokes in the active port exceeded 50% of all pokes made during that sequence. This ensures that the behavior is not random and reflects meaningful engagement with the task. In our data, 14924 of 17903 (83.36%) meals exceeded 50% overall accuracy. Additional meal statistical properties are available in Tables 1 and 2.

**Table 1:** Statistics of meals for groups and session types.

Session Type	Group	Number of mice	Total number of meals	Mean pellets per meal	SD pellets per meal
FR1	CASK-KD	31	2049	2.535060137	0.310982681
FR1	Control	35	2115	2.456629232	0.32710205
FR1	Validation	18	788	2.188199348	0.128855362
REV	CASK-KD	24	3987	2.877819391	0.27690784
REV	Control	27	4007	2.64796773	0.319282141
REV	Validation	18	2371	2.393708662	0.147061723

**Table 2:** Distribution of meals size for groups and session types.

Session Type	Group	2-pellets	3-pellets	4-pellets	5-pellets	6-pellets	7-pellets	8-pellets
FR1	CASK-KD	1167	697	155	21	6	3	0
FR1	Control	1306	713	79	10	5	1	1
FR1	Validation	634	147	6	1	0	0	0
REV	CASK-KD	1435	1738	712	91	10	1	0
REV	Control	1914	1757	290	29	12	4	1
REV	Validation	1547	724	97	3	0	0	0

#### Reversal task behavioral block

During reversal tasks, the FED3 device alternated the designated active poke after a specified number of pellet retrievals (25). Thus, a block encompasses all events from immediately after one active-poke switch until just before the next switch.

#### First meal time in each behavioral block

Quantified the latency from the start of the current behavioral block to the start of its first meal. Meals that began before the start of the current behavioral block were excluded to ensure each measurement reflected new conditions post-switch. This measure was analyzed both in absolute terms (minutes since behavioral block start) and as a normalized proportion relative to the block duration.

### Win-Stay/Lose-shift and strategy evolution analysis

A ‘win’ was defined as a mouse poking the active port rewarded by a pellet, while a ‘loss’ was defined as poking the inactive port. The Win-Stay probability was calculated as the proportion of trials where a mouse kept poking the active port following a ‘win’ (P(stay | win)). The lose-shift probability was calculated as the proportion of trials where a mouse switched to poking the active port following a ‘loss’ (P(shift | lose)). Dynamics of decision-making strategies within reversal blocks were assessed by calculating the win-stay and lose-shift probabilities overall and at two distinct phases: the initial (first 10 pellets) and late (last 10 pellets) phases of each reversal block. When computing by phase, the final block of each session was excluded to ensure that the analysis included complete behavioral sequences.

### Accuracy measurement of a given FED3 data

For a given FED3 dataset, the accuracy was calculated as the number of nose pokes in the active port divided by the total number of nose pokes in both the active and inactive ports.

### 80% accuracy milestone during FR1 task

The 80% accuracy milestone marked the time when a mouse first achieved and sustained a high level of accuracy, specifically maintaining an accuracy rate above 80% for at least two consecutive hours.

### Learn score % during a behavioral block

The ‘learn score’ % was calculated as the accuracy in the first % nose poke actions with no pellet available (nose poke in either active or inactive port). For example, Learning Score 25% was the accuracy as measured over the first 25% of nose poke actions in a given behavioral block. This measurement was invariant to the length of the behavioral block.

### Learn result during a behavioral block

The ‘learn result’ % was calculated as the accuracy of the last 25% of nose poke actions with no pellet available (nose poke in either active or inactive port).

### Unsupervised Learning and Generalization of meal accuracy pattern

Meal accuracy patterns were used to classify them into high-accuracy and low-accuracy, using the following pipeline:

1. Meals containing 3–5 pellets were selected, and their accuracy patterns were calculated.
2. Accuracy patterns of meals, having the same pellet length, were clustered using K-means, where the number of clusters (K) was determined using the elbow method and Silhouette score. Then, clusters were manually identified as representing either high accuracy or low accuracy.
3. Subsequently, two different neural network models (differing by architecture, see below) were trained on the labeled accuracy patterns from the k-means to obtain a robust classification and remove non-generalizable patterns produced by the K-Means clustering.
4. The trained models were then evaluated for their performance. After achieving the desired performance, the models were used to classify meals across all experiments.

### CNN-based model

The CNN-based model consisted of two convolutional blocks. The first block included a convolutional layer with 16 channels, and the second block included a convolutional layer with 32 channels. Both layers use a kernel size of 2 and were followed by ReLU activation functions. A MaxPooling layer was applied after each convolutional block, and the final feature map was passed through a fully connected layer to map the extracted features to two output classes, representing high-accuracy and low-accuracy meals.

### LSTM-based model

The LSTM-based model was comprised of two LSTM layers with a hidden dimension of 400, followed by a fully connected layer that mapped the output to two classes, representing high-accuracy and low-accuracy meals.

Both models were trained using a dataset derived from the k-means labeled meal accuracy patterns. The dataset was 90/10 train-test split and the models were trained with a batch size of 256 and Adam optimizer. The learning rate of the LSTM-based and CNN-based model was 0.0001 and 0.001, respectively. Both models were trained for 50 epochs. The LSTM-based model achieved ~99% testing accuracy with ~0.99 F1 Score, while the more efficient CNN-based model achieved ~98.5% testing accuracy and ~0.98 F1 Score. The classification errors were driven by the edge cases caused by the clustering process. For reference, we spent about 20 min performing K-Means-assisted data annotation. Therefore, we believe the total time to develop and test a deep learning-based algorithm was shorter than or equal to that for a rule-based algorithm, especially when we need to detect heterogeneous time-series structures or have multiple target classes.

### Correlation analysis of behavioral metrics

Pairwise Pearson correlation coefficients (r) were computed between the following behavioral metrics: Reversal block overall Win-stay probability, Reversal block overall lose-shift ratio, learning result (accuracy in the final 25% of blocks), overall session accuracy, latency to the first accurate meal (defined by ML-classification), and the ratio of time to the first ‘accurate’ meal relative to block duration. Values for the behavioral features were obtained from mice across all experimental groups. The pairwise Pearson correlation coefficients were represented in a single correlation matrix.

### Statistical analysis

An independent two-tailed t-test was used for all statistical tests, implemented using the SciPy package. In all the figures, box plots show the median, 25, and 75% percentiles, with error bars reflecting the standard error of the mean.

## Results

### Development of a FED3-based behavioral paradigm and analysis pipeline to assess cognitive flexibility

**Fig. 1:**
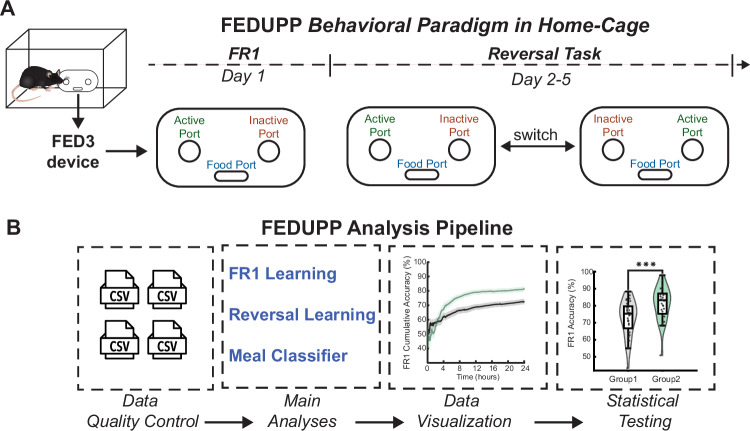
Schematic of FEDUPP Behavioral Assay and Analysis. **A** Timeline and schematic of the home-cage behavioral paradigm. **B** Schematic of the FEDUPP analysis pipeline.

To assess cognitive flexibility in a home cage environment, FED3 devices [26] were used to monitor mouse food intake over a two-phase behavioral paradigm. In the first phase, mice performed a nose-poke-based two-alternative forced-choice task with a fixed ratio of one nose poke per reward (FR1) for approximately 24 h. In the second phase, mice completed a reversal version of the same two-alternative forced-choice task for approximately 72 h (Figs. 1, S2), in which the active port switched after every 25 pellets collected (Figs. 1, S2). The interval between port switches, defined by the time taken to collect 25 pellets, was termed a block. An in-house analysis pipeline, FEDUPP, was developed to extract learning and cognitive flexibility metrics from FED3 data. FEDUPP quantifies performance across multiple timescales, including overall accuracy, 80% accuracy milestone, and meal pattern. ‘Accuracy’ was defined as the proportion of correct (active) port pokes relative to all port pokes (active + inactive), within a defined time window (FR1 session, behavioral block, meal) (Fig. S3, see Methods). The ‘80% accuracy milestone’ was defined as the earliest time point at which accuracy exceeds 80% for a sustained 2-h window, providing a shorter time scale measure of learning independent of the length of the entire session (Fig. S3, see Methods). In addition, sequences of pellet collection events were identified as meals, and their measured accuracy was the meal’s accuracy (Fig. S4, see Methods). A long short-term memory (LSTM)-based classifier was trained on meals and their accuracy collections to classify meals into high-accuracy and low-accuracy. The occurrence of the first high-accuracy meal was an additional evaluation of learning (Fig. S4, see Methods).

A group of wild-type (WT) C57/Bl6 mice was used for initial method development. This group enabled the establishment of baseline performance metrics and refinement of computational analysis tools.

### Mice acquired above chance-level FR1 performance in less than 24 h

**Fig. 2:**
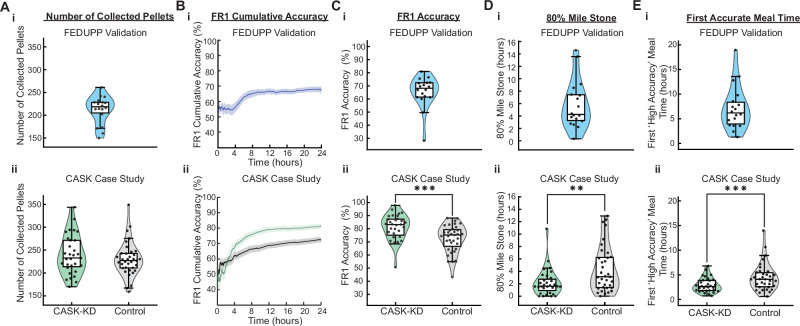
FR1 performance metrics. **A** Total number of pellets collected during the FR1 session for the WT validation group (Ai), CASK-KD and Control groups (Aii). **B** Groups dynamics of accuracy during the FR1 of the WT validation cohort (Bi), and CASK-KD and Control cohorts (gray- Control, CASK-KD - green) (Bii) during the 24 h of the FR1 task. **C** Overall accuracy during the FR1 session for the WT validation group (Ci), and CASK-KD and Control groups (Cii). **D** Time to reach 80% accuracy in a time window of two hours for the WT (Di), CASK-KD and Control (Dii) groups during FR1. **E** Time to first accurate meal during the FR1 for the WT (Ei), and CASK-KD and Control (Eii) groups. Asterisks denote between-group differences (**p < 0.01, ***p < 0.001, two-tailed unpaired t-test). In panels Bi and Bii, grey and green colors designate Control and CASK-KD groups, respectively.

In the FR1 session, WT mice collected an average of ~212 pellets (212.10 ± 6.89, *N* = 18, Fig. 2Ai), corresponding to ~4.2 g of chow. The mice improved their accuracy over time, reaching an average overall accuracy of ~65% (65.28 ± 1.8%, *N* = 18, Fig. 2Bi, Ci) within the initial 24 h. Notably, the 80% accuracy milestone was achieved after the first ~5 h (5.77 ± 0.88 h, *N* = 18, Fig. 2Di) from the start of the FR1. Moreover, using the pre-trained LSTM-based meal classifier (Fig. S3), the first high-accuracy meal was detected at ~7.1 h (7.13 ± 1.01, *N* = 18, Fig. 2Ei).

When applied to the CASK-KD and Control mice, the FR1 paradigm revealed that the CASK-KD group showed a non-significant trend toward collecting more pellets than Control group (CASK-KD: 239.81 ± 7.51, *N* = 31; Control: 230.08 ± 6.16, *N* = 35, Fig. 2Aii). Compared to cControl mice, the CASK-KD mice exhibited significantly higher overall accuracy (CASK-KD: 81.225% ± 1.72, *N* = 31; Control: 72.69% ± 1.76, *N* = 35; *p* = 0.001, Fig. 2Bii, Cii) and required significantly less time to reach the 80% milestone (CASK-KD: 2.17 ± 0.38 h, *N* = 31; Control: 4.39 ± 0.62 h, *N* = 35; *p* = 0.005, Fig. 2Dii). In addition, the first high-accuracy meal was achieved faster by the CASK-KD compared to the Control group (CASK-KD: 2.92 ± 0.26 h, *N* = 31; Control: 4.50 ± 0.47 h; *N* = 34, there was 1 significant outlier (>20 h in Control), *p* = 0.007, Fig. 2Eii).

Across all groups, average performance exceeded chance level by the end of the FR1 phase, with CASK-KD mice showing an increased accuracy and faster acquisition relative to controls, as reflected in the 80% milestone metric.

### Mice perform a reversal paradigm with above-chance accuracy during each block

Following the FR1 assay, mice completed a reversal task lasting at least 72 h (Figs. 1A, S2. In the reversal task, the active port switched every 25 pellets collected, thus creating a new behavioral block. Performance within blocks was quantified using two FEDUPP-derived metrics (Fig. S5). The first metric, ‘learn score’, measured cumulative accuracy within a block, calculated as the proportion of correct (active port) pokes from block onset up to each nose-poke event. This measure was time-invariant and reflected the progressive acquisition of the correct port over the course of a block. The second metric, ‘learn result’, calculated accuracy over the final 25% of pokes in a block, providing a snapshot of end-of-block performance once adaptation had occurred.

**Fig. 3:**
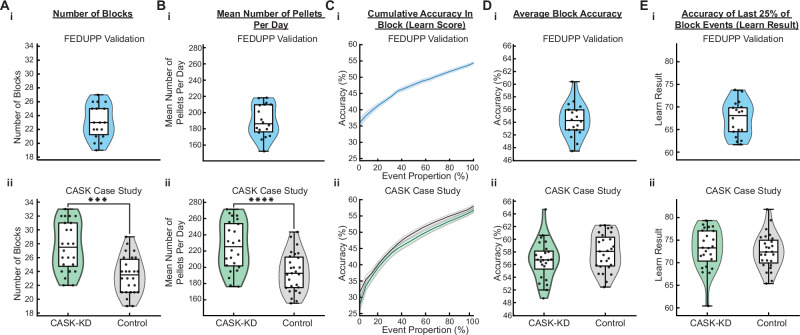
Reversal task block performance metrics. **A** Total number of blocks completed during the reversal task for the WT (Ai), and CASK-KD and Control (Aii) groups. **B** Average number of daily collected pellets during a reversal task for the WT (Bi), and CASK-KD and Control (Bii) groups. **C** Cumulative accuracy (learn score) as a function of event proportion within a block, shown as mean ± SEM, for the WT group (Ci) and CASK-KD and Control cohorts (gray- Control, CASK-KD - green) (Cii). **D** The average total accuracy per-block for WT (Di) and CASK-KD and Control (Dii) groups. **E** Learn Result (accuracy over the final 25% of pokes per block) for the WT (Ei), and CASK-KD and Control (Eii) groups. Asterisks denote significant between-group differences (***p < 0.001, ****p < 0.0001; two-tailed unpaired t-test). In panels Ci and Cii, grey and green designate Control and CASK-KD groups, respectively.

The WT group showed that, over the 3-day reversal phase, mice completed an average of ~23 (23.11 ± 0.56, *N* = 18, Fig. 3Ai) blocks, during the 72 h of the task, and collected an average of ~192 pellets per day (192.57 ± 9.11, *N* = 18, Fig. 3Bi). The ‘learn score’ progressively increased with the number of nose pokes the mouse performed during a block (Fig. 3Ci), resulting in total accuracy for a block being just above chance level (51.03 ± 0.65%, *N* = 18, Fig. 3Di). The ‘learn result’ averaged ~67% (Fig. 3Ei, 67.41 ± 0.87%, *N* = 18), well above the chance level.

When the same analyses were applied to the CASK-KD and Control groups, CASK-KD mice completed significantly more blocks during the reversal task (27.54 ± 0.71 vs 23.46 ± 0.53, CASK-KD *N* = 24, Control *N* = 27, *p* = 0.036, Fig. 3Aii) and collected more pellets per day when compared to the Control group (225.55 ± 5.5 vs 193.32 ± 4.2, CASK-KD *N* = 24, Control *N* = 27, *p* = 2.83*10^{-5}, Fig. 3Bii). Despite these increases, the dynamics of the ‘learn score’ metric were similar between groups (Fig. 3Cii) with total accuracy for a block being just above chance level (56.71 ± 0.62% vs 57.77 ± 0.54%, CASK-KD *N* = 24, Control *N* = 27 *p* = 0.215 Fig. 3Dii). Similarly, both groups achieved a ‘learning result’ of ~72% on average (CASK-KD: 73.03 ± 0.90% ; Control: 72.49 ± 0.73; CASK-KD *N* = 24, Control *N* = 27, *p* = 0.601, Fig. 3Eii). This analysis indicated that all groups consistently performed above chance level within each reversal block, and that the CASK-KD and Control groups had similar performance, as measured by these behavioral metrics.

### Analysis of meal structure during the reversal block reveals that high-accuracy meals appear in reversal blocks

**Fig. 4:**
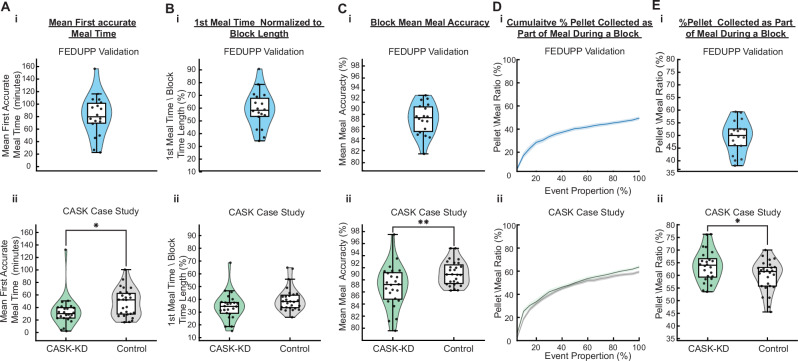
Statistics of meal properties during the reversal task blocks. **A** Average time of first meal during a reversal task block (as classified by the LSTM-based meal classifier) for the WT (Ai), and CASK-KD and Control (Aii) groups. **B** Average time of first meal during a reversal task block normalized to the block duration for the WT (Bi), and CASK-KD and Control groups (Bii). **C** Average meal accuracy during the block for the WT (Ci), and CASK-KD and Control (Cii) groups. **D** Cumulative proportion of pellets collected within meals as a function of block progression (the percentage of pellets collected during a block), shown as mean ± SEM, for the WT (Di), and CASK-KD and Control groups (gray- Control, CASK-KD - green) (Dii). **E** Mean ratio of pellets collected as part of a meal relative to the total number of pellets collected per block for the WT (Ei), and CASK-KD and Control (Eii) groups. Asterisks denote significant between-group differences (*p < 0.05, **p < 0.01; two-tailed unpaired t-test). In panels Aii-Eii, grey and green designate Control and CASK knockdown groups, respectively.

To determine whether structured feeding patterns emerged during the reversal task and whether these patterns reflected task accuracy, meal analysis was applied as described in Fig. S4 (see Methods). Across all groups, a meal was detected in >98% of reversal blocks, indicating that goal-directed feeding events were preserved during reversal performance. In the WT group, the time required to reach the first high-accuracy meal from the start of the reversal block was ~81.6 min (81.66 ± 7.51 min, *N* = 18, Fig. 4Ai). When this time was normalized to the duration of each block, the mean ratio was approximately ~59% of the block length (58.9 ± 3.3%, *N* = 18, Fig. 4Bi). In addition, the mean accuracy within these meals was ~89% (89.19 ± 0.6%, *N* = 18, Fig. 4Ci). The proportion of pellets consumed as part of a meal increased as the block progressed (Fig. 4Di**)**, reaching an average of ~49% (49.4 ± 1.5%, *N* = 18, Fig. 4Ei). This indicated that about half of the pellets were collected during meals.

Comparison of the CASK-KD and the Control groups revealed that the time required for the first accurate meals, was shorter for the CASK-KD (CASK-KD: 33.35 ± 4.93 min, *N* = 24; Control: 47.50 ± 4.21 min; *N* = 27; *p* = 0.036, Fig. 4Aii). When normalized to the length of a block, CASK-KD group trended toward collecting their first ‘high accuracy’ meal earlier in the block than Control group (CASK-KD: 34.4 ± 2.2%, *N* = 24; Control: 40 ± 1.8%, *N* = 27; *p* = 0.051, Fig. 4Bii). In addition, the CASK-KD group had a lower meal accuracy compared to the Control group (CASK-KD: 88.93 ± 0.7, *N* = 24; Control: 91.09 ± 0.38%, *N* = 27, *p* = 0.008, Fig. 4Cii). Both groups showed an increase in the proportion of pellets consumed as part of a meal as the block progressed (Fig. 4Dii). However, the CASK-KD group reached a significantly higher average than the Control with ~63% and ~59%, respectively (CASK-KD: 63.6 ± 1.3%, *N* = 24; Control: 59.4 ± 1.1%, *N* = 27, *p* = 0.0197, Fig. 4Eii).

These findings indicate that reversal blocks are characterized by structured, accurate feeding bouts and that CASK-KD mice, while ultimately achieving similar meal accuracy, still initiate ‘high accuracy’ meals earlier with a higher number of pellets consumed as part of the meal.

### Comparison of FEDUPP metrics with win-stay/lose-shift

**Fig. 5:**
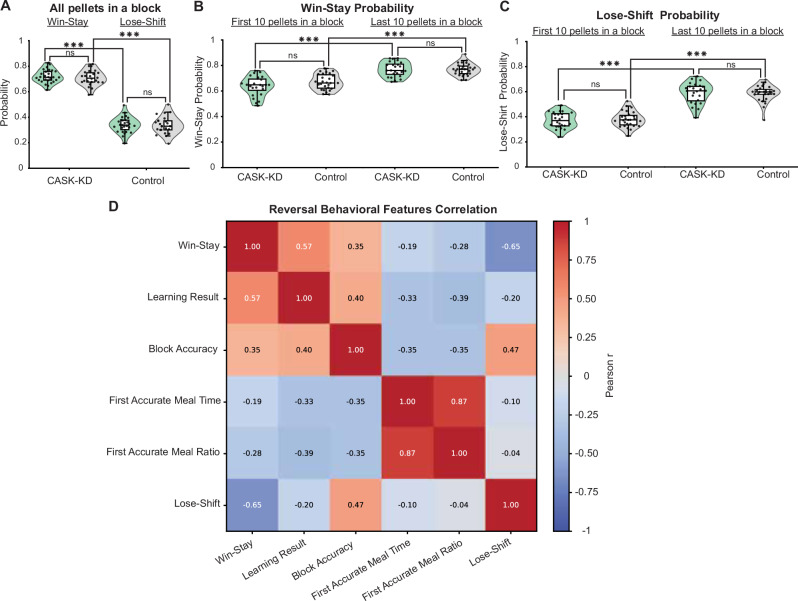
Comparison of Win-Stay/Lose-Shift to reversal block metrics. **A** Win-stay/Lose-Shift probabilities of CASK-KD and Control groups averaged across all reversal blocks. ***p < 0.0001 (two-way ANOVA with post-hoc correction). **B, C** Win-stay/Lose-Shift probabilities of CASK-KD and Control groups as a function of the reversal block phase, early (B, first 10 pellets, left side) or late (C, last 10 pellets, right side). ***p < 0.001(two-way ANOVA with post-hoc correction). **D** Correlation matrix of Pearson correlation coefficients between all the reversal block behavioral metrics across all subjects. In panels A and B, grey and green designate Control and CASK knockdown groups, respectively.

Next, the win-stay/lose-shift, a known behavioral strategy when animals need to shift choices [28], was calculated during reversal blocks. The analysis of win-stay/lose-shift across all blocks showed that mice from both groups had a significantly higher win-stay than lose-shift during reversal blocks (main effect of strategy, Two-way ANOVA: F(1, 98) = 989.99, *p* < 0.001). This observation was confirmed for both groups, CASK-KD and Control, using post-hoc comparisons (CASK-KD (*N* = 24): win-stay 0.709 ± 0.012 vs. lose-shift 0.339 ± 0.014, *p* < 0.001, Control (*N* = 27): win-stay 0.720 ± 0.010 vs. lose-shift 0.340 ± 0.02, *p* < 0.001, Fig. 5A). Importantly, no significant effect of group (*p* = 0.595) or group x strategy interaction (*p* = 0.688) was observed. Next, the win-stay/lose-shift (strategy) probabilities were compared between the initial (first ten pellets, Fig. 5B) and late (last ten pellets, Fig. 5C) stages of the block(phase). Mice from both groups exhibited a marked increase in win-stay and lose-shift probability from the initial and late phase of the reversal block (Two-way ANOVA, main effect of window; Win-stay: F(1, 98) = 90.81, *p* < 0.001; Lose-shift: F(1, 98) = 242.16, *p* < 0.001; CASK-KD x Win-Stay (*N* = 24): 0.64 ± 0.02 to 0.77 ± 0.01, Post-hoc corrected *p* < 0.001, Control x Win-Stay (*N* = 27): 0.67 ± 0.01 to 0.77 ± 0.01 and CASK-KD x lose-switch (*N* = 24): 0.37 ± 0.01 to 0.59 ± 0.02, Post-hoc corrected *p* < 0.001, Control x lose-switch (*N* = 27): 0.38 ± 0.01 to 0.59 ± 0.01, Post-hoc corrected *p* < 0.001, Fig. 5B, C). Thus, win-stay/lose-shift probabilities, whether calculated across the entire reversal block or in the initial and late stages of the block, did not differ between the Control and CASK-KD groups.

The correlation matrix between all of the behavioral metrics (Fig. 5D), calculated for mice from all of the study groups, showed that the win-stay and lose-shift probabilities were positively correlated with the reversal block accuracy (r = 0.35 and r = 0.47 respectively, Fig. 5D). Conversely, the ‘learn result’ was positively correlated with the win-stay probability and negatively correlated with lose-shift probability (r = 0.57 and r = −0.2, respectively, Fig. 5D). In addition, the lose-shift probability showed a negative correlation with the win-stay probability (r = −0.65, Fig. 5D). Taken together, the win-stay and lose-shift probabilities calculated across the reversal blocks didn’t distinguish between the Control and Cask-KD groups and showed correlations with the previously presented behavioral metrics.

## Discussion

FED3 devices were previously used to study feeding behavior [26] and, more recently, to assess cognitive flexibility either outside of the homecage sessions [29, 30] or for limited home-cage sessions [31]. The present study introduces two main innovations. First, a fully home-cage, two-phase paradigm was developed to continuously measure learning and behavioral flexibility by using FED3 located in the mouse homecage. In the first phase, which lasted a day, mice acquired an FR1 task, as indicated by their accuracy above chance level in selecting the active port, on average. In the second phase, the mice performed a reversal task lasting 3 days, in which the active port was switched every 25 collected pellets; thus, the reversal frequency was contingent on the mouse’s actions. Second, an open-source analysis pipeline, FEDUPP, was developed to extract multi-timescale measures of learning and cognitive flexibility from the FED3 data, including novel metrics of accuracy milestone and meal-based performance. Together, these advances allow minimally invasive, continuous tracking of operant learning and reversal behavior under naturalistic conditions.

### Estimating learning at different time scales

FEDUPP assumed that mouse FED3 nose poking represents a trial structure in which the action of a nose poke in the active port results in a pellet being released and the port becoming active again after the pellet was collected. This structure creates a continuous, self-initiated trial format without explicit cues, approximating naturalistic decision-making. Behavioral paradigms that measure learning and behavioral flexibility, based on consecutive trials performed by the animal, tend to run into the fundamental question “did the animal learn the task contingency?”, particularly in self-paced reversal tasks where block duration is determined by the animal’s behavior; a topic still debated [32–34].

To address this, FEDUPP introduced both coarse and fine temporal metrics. The overall accuracy captured performance across an entire session or block, which is a standard metric in the field [34]. The ‘80% accuracy milestone’ was designed to estimate learning on a shorter time scale. This metric used a moving window of predefined time length (2 h) to calculate accuracy, identifying the earliest point at which accuracy exceeds 80% within that window. This approach is conceptually similar to methods that count consecutive correct choices in a window composed of a predefined number of trials [34, 35], but with two key differences. First, FEDUPP applies a time-based window rather than a fixed trial count, accommodating a temporal variable based on the variable pace of self-initiated responding in the home cage. Second, the method applied a looser requirement for consecutive correct responses and did not conduct a formal statistical test of a null hypothesis for learning within the defined time window. This makes the metric flexible and well-suited for continuous, self-paced behavioral datasets where trial structure is not externally imposed.

### Meal-based analysis as a marker of goal-directed exploitation behavior

During a behavioral task, mice adopt strategies shaped by the outcomes of their actions [36–38]. These strategies are dynamic, with individual mice shifting between exploration and exploitation during trials of a given session [39, 40] based on internal states [41] and external factors [13, 42]. In the FR1 and reversal tasks, two features may influence a bias toward more exploration based strategies: (1) there is no cost for poking the inactive port, and (2) the effort required to obtain a pellet (a single nose poke) is a relatively low value from an ethological perspective [43].

In this assay, a higher exploratory/ reduced exploitation strategy could lead to more random poking of the active and inactive ports, leading to an increase in inactive port poking over time and apparent reduced accuracy of mouse performance. To more precisely discern between exploration and exploitation, we focused on patterns of pellet collection that can be classified as meals, which represent a motivated, increased exploitation strategy [44]. In fact, homeostatic drive increases the motivational salience of food and enhances goal-directed exploitation responding, as shown by increases in progressive-ratio breakpoints, activation of valuation circuits during feeding [45].

Pellets collected within 1 min were classified as part of a meal if the accuracy was above 50%. In the reversal task, roughly 50–60% of pellets were collected within these meal bouts. We then defined meal accuracy as the proportion of correct nose-pokes during a meal and employed a binary classifier (see Methods) to label each meal as high-accuracy or low-accuracy. Since feeding motivation drives exploitation over exploration, meal accuracy is a sensitive proxy for task understanding. Focusing on accuracy during meals yields a more fine-grained estimate of task contingency ‘understanding’ than session-wide accuracy. For this reason, FEDUPP includes metrics such as the first ‘high accuracy’ meal, which can serve as a better measure of how quickly the mouse has acquired the task. Therefore, a high overall accuracy along with a short time to first ‘high accuracy’ meal suggests rapid task acquisition and consistent goal-directed responding, whereas high overall accuracy with a long time to first high-accuracy meal may indicate a slower engagement in goal-directed feeding despite correct responses elsewhere. Low meal accuracy suggests persistent exploratory behavior or weak task knowledge during motivated feeding, whereas changes in meal accuracy across blocks may reflect changes in motivation. Therefore, FEDUPP allows researchers to use meal-based metrics alongside standard accuracy measures to disentangle motivation-driven changes from true cognitive flexibility deficits.

### Effect of CASK knockdown on cognitive flexibility

CASK is a multi-domain scaffolding protein enriched at synapses and implicated in synaptic organization, neurotransmitter release, and plasticity [46, 47]. Knockdown of CASK in the dorsal hippocampus may alter synaptic signaling required for the timely incorporation of new contextual information into ongoing behavior.

In the FR1 phase, the CASK-KD mice reached the 80% accuracy milestone more quickly and achieved higher overall accuracy, suggesting increased exploitation behavior once the active port was identified. In this context, increased accuracy can be explained as reduced exploration and increased exploitation. In the reversal task, CASK-KD mice achieved the same accuracy as the Control group but were faster when collecting the first accurate meal. In other words, the reduced exploration that may have enhanced FR1 performance did not delay recognition of the port-role change, but resulted in a faster onset of ‘high-accuracy’ meals despite similar overall accuracy. This suggested that CASK KD in the dorsal hippocampus alters the dynamics of adapting to new contingencies without impairing the capacity to learn them.

This interpretation aligns with prior work showing that manipulations of the dorsal hippocampus can improve instrumental performance metrics through complex behavioral changes, such as reduced behavioral flexibility and/or altered motivation, rather than improved learning per se [48, 49]. Importantly, our data indicate that CASK is not a limiting factor for hippocampal-dependent updating of task rules or flexible goal-directed learning, but rather influences the temporal dynamics with which newly acquired information is translated into stable behavioral responses.

### FEDUPP behavioral metrics in relation to win-stay/lose-shift probabilities

The win-stay/lose-shift analysis showed a consistent decision strategy for both Control and CASK-KD mice. Mice in both groups displayed significantly higher win-stay than lose-shift probabilities throughout reversal blocks. When win-stay/lose-shift probabilities were examined across the initial and late stages of reversal blocks, both groups exhibited a marked increase in win-stay and lose-shift probabilities. This temporal progression indicates that, in the early phase of a block immediately following an active poke switch, mice were less likely to shift after an error and tended to perseverate on the previously rewarded port. By the end of the block, however, error sensitivity had increased, and mice more reliably shifted following negative feedback or sustained behavior following positive feedback. The CASK-KD group showed comparable strategy shifts to Control group, suggesting stable flexibility and learning on the reversal task. The absence of any group differences in win-stay/lose-shift probabilities stand in contrast to the significant group differences observed in the FEDUPP meal-based metrics. CASK-KD mice showed a faster onset of the first high-accuracy meal and a higher proportion of pellets consumed within meals, yet employed an identical decision strategy to controls. This discrepancy suggests that dorsal hippocampal CASK knockdown alters the temporal dynamics of goal-directed behavior without affecting the underlying decision strategy. Win-stay/lose-shift analysis, while capturing trial-by-trial decision strategy, does not detect this difference, because the difference lies not in how mice decide on each poke, but in when structured, motivated feeding sequences emerge within a block.

The correlation analysis provides further insight into the relationship between decision strategies and behavioral performance. Win-Stay probability was positively correlated with both Learning Result and Overall Accuracy, confirming that the ability to sustain a rewarded action is a primary determinant of high reversal performance. Conversely, the Lose-Shift strategy showed a moderate negative correlation with the Win-Stay strategy and a weak negative correlation with Learning Result. This might imply that high-performing mice rely predominantly on exploiting known rewards (high Win-stay) rather than exploring after errors (high Lose-shift). It is also worth noting that the Lose-shift probability is generally less than 50% across all mice, which might suggest poor flexibility. Notably, the strong negative correlation between Win-Stay and Lose-Shift suggests a potential trade-off in cognitive strategies. Mice that rigidly adhere to a “stay” strategy may be less sensitive to errors, whereas those that shift frequently after losses may struggle to stabilize their behavior on the active port. Importantly, the meal-based metrics showed near-zero correlations with both win-stay and lose-shift probabilities, indicating that meal-based metrics capture a dimension of reversal behavior that is statistically independent of trial-by-trial decision strategy and reflects the emergence of structured, motivated feeding sequences. Taken together, these findings support the view that FEDUPP meal metrics provide complementary and non-redundant information relative to established reversal learning measures, capturing aspects of goal-directed exploitation behavior that standard strategy analyses do not assess.

### Limitations of the paradigm and study

One of the hallmarks of learning tasks that aim to measure the cognitive abilities in mice is their reliance on a motivational state to drive task acquisition. In the present paradigm, hunger served as the primary motivational state driver of the mouse’s engagement and performance. In the presented study, the CASK-KD mice consumed more pellets in FR1 than the Control mice. The current paradigm design and analysis cannot distinguish whether this difference reflects increased hunger or altered metabolic state, or changes in cognitive processes or motivation that influence task performance [43]. The behavior assay accompanying FEDUPP may integrate, in future iterations, such methods to improve the interpretability of behavioral differences.

### Future directions

This study assessed learning and cognitive flexibility in mice using continuous, food acquisition-based tasks performed in their home cage, and introduced FEDUPP, an open-source software package for analyzing FED3 data. Future work could expand in two main areas. First, the behavioral assays will be expanded by varying the duration and/or the number of reversal blocks in the reversal task to examine how task structure influences mouse performance. Second, FEDUPP will include a graphical user interface tool, enabling users to easily browse FED3 datasets, define custom metrics, such as accuracy milestones, specify meal parameters, and choose visualization formats. Furthermore, expansion of the analysis tools will support more advanced methods to assess learning and cognitive flexibility.

## Supplementary information

Supplementary Figure Legends

Supplementary Figure 1

Supplementary Figure 2

Supplementary Figure 3

Supplementary Figure 4

Supplementary Figure 5

## Data Availability

FED3 behavioral data is available on https://github.com/ftlabucsd/FEDUPP.
