# Supplementary material for: The development of FEDUPP: feeding experimentation device users processing package to assess learning and cognitive flexibility
Source: Transl Psychiatry. 2026 May 16;16:348. doi: 10.1038/s41398-026-04091-6 (PMC13346605; doi:10.1038/s41398-026-04091-6)
Supplement: Supplementary file 1 — Supplementary Figure Legends [file 41398_2026_4091_MOESM1_ESM.docx]

Supplementary Figure Legends

**Figure S1. Verification of dorsal hippocampal CASK knockdown. A.** Relative Cask mRNA expression normalized to Actb in N2a cells transfected with shCASK or shScramble constructs, measured by RT-qPCR. Data is shown as mean ± SD of 3 biological replicates, ** p<0.01 (unpaired t-test). **B.** Representative coronal brain section showing GFP expression (green) in the dorsal hippocampus following AAV injection, counterstained with DAPI (blue). Atlas overlay indicates injection site targeting.

**Figure S2**. **State machine schematics of the FR1 and reversal tasks.**  **Upper panels,** state machine diagrams describing the event logic of both the FR1 (left) and reversal (right) tasks. Color-coded labels denote events logged by the FED3 device: active port poke (green), inactive port poke (orange), and pellet collected (purple). **Lower panel,** a schematic of the logged events is visualized as a time series.

**Figure S3. Schematic of FEDUPP FR1 performance metrics**. **Upper panel,** given a time series of ‘active’ and ‘inactive’ port pokes (green and orange, respectively), the accuracy between time points t and t' is calculated as active port pokes divided by the total number of port pokes in that interval. **Middle panel,** cumulative accuracy at time point t is calculated as the accuracy from session onset ( 0) to t, where t is smaller than a given maximal value T. **Lower panel,** the 80% accuracy milestone is calculated the earliest time point t at which accuracy in the time range t to t+tau (tau length of time window) is bigger than 80%, where τ is a fixed window length of 2 hours.

**Figure S4 - Schematic of FEDUPP meal metrics** - **Upper panel,** given a time series of ‘active’, ‘inactive’ port pokes and ‘pellet collected’ events (green, orange and purple respectively), meal is defined as a series ‘pellet collected’ events in which the sum of time difference between them is smaller than a maximum defined by Tmeal. **Middle panel**, meal accuracy is calculated as the accuracy based on the active and inactive port poke events occurring during a meal. **Lower panel**, a meal is classified as either accurate or inaccurate based on an LSTM-based classifier trained on meal accuracy patterns.

**Figure S5. Schematic of metrics calculated by FEDUPP based on time-invariant nose poking events.** **Upper panel,** the temporal series of ‘active’ and ‘inactive’ port pokes (green and orange, respectively) with total N events between 0 and T, was converted to an N-length vector, which is time invariant. **Middle panel** (Upper) The learn score is the cumulative accuracy from event 1 to event K, plotted as a function of K divided by N (event proportion, x-axis). (Lower) The learn result is defined as the accuracy over the final 25% of poke events in a block (events K to N, where (N−K)/N ≈ 0.25), providing a measure of end-of-block performance.
