# Supplementary figures and images for: The development of FEDUPP: feeding experimentation device users processing package to assess learning and cognitive flexibility
Source: Transl Psychiatry. 2026 May 16;16:348. doi: 10.1038/s41398-026-04091-6 (PMC13346605; doi:10.1038/s41398-026-04091-6)

A

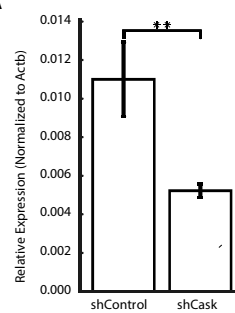

B

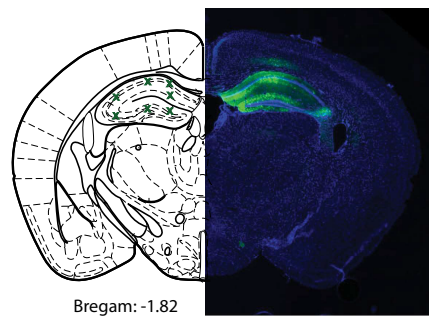

Supplement: Supplementary file 2 — Supplementary Figure 1 [file 41398_2026_4091_MOESM2_ESM.pdf]

FR1 - 1 Day

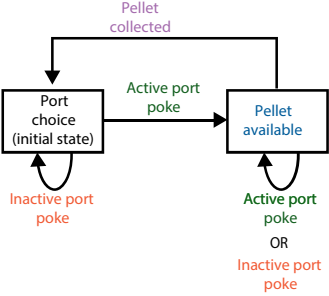

Reversal Task - 3 Days

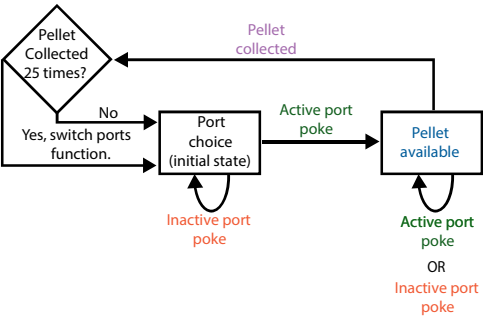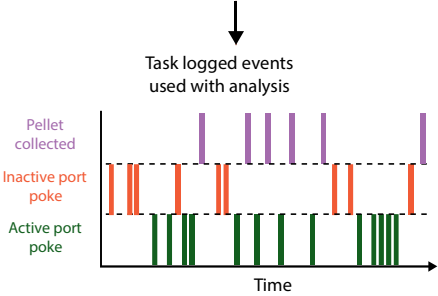

Supplement: Supplementary file 3 — Supplementary Figure 2 [file 41398_2026_4091_MOESM3_ESM.pdf]
