# Supplementary material for: The development of FEDUPP: feeding experimentation device users processing package to assess learning and cognitive flexibility
Source: Transl Psychiatry. 2026 May 16;16:348. doi: 10.1038/s41398-026-04091-6 (PMC13346605; doi:10.1038/s41398-026-04091-6)

FR1 Performance Metrics

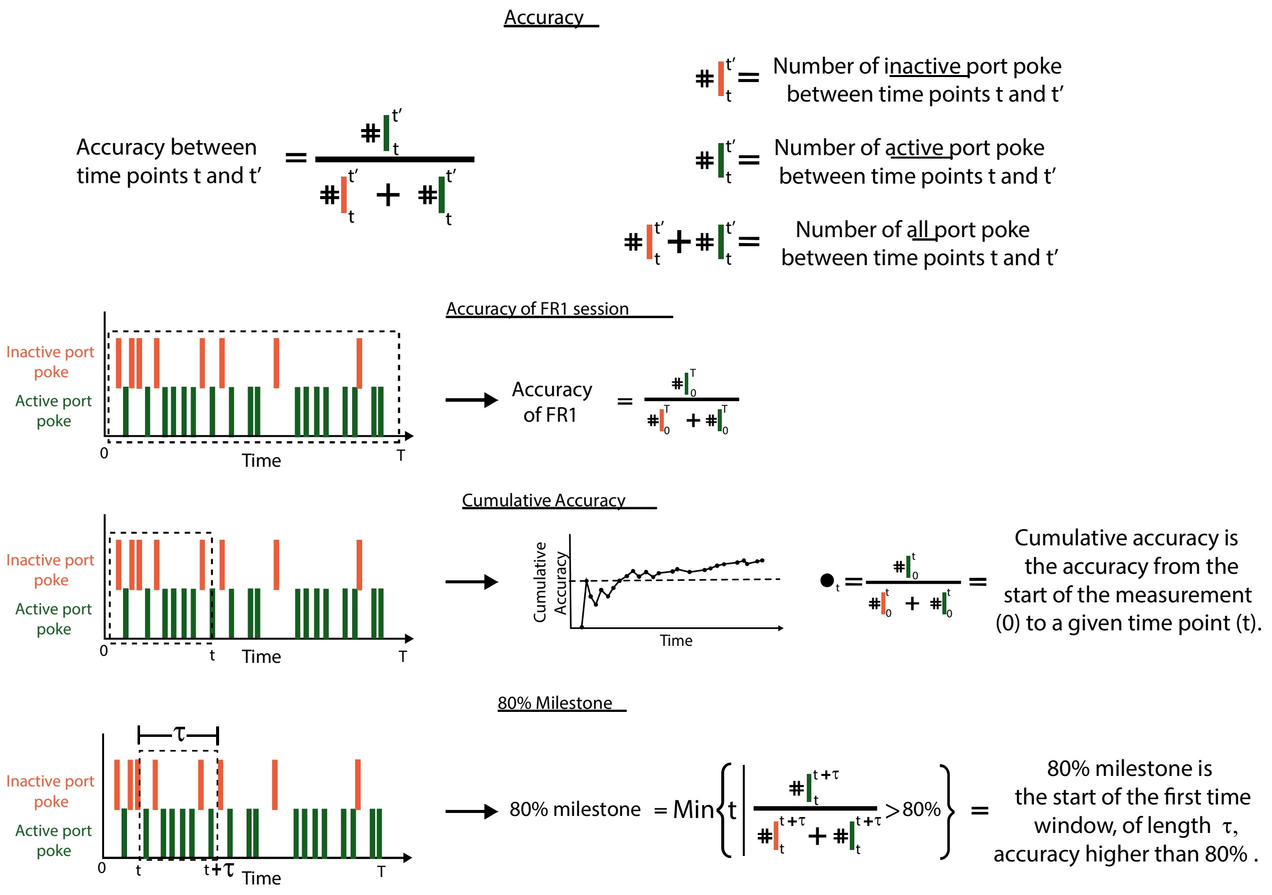

Supplement: Supplementary file 4 — Supplementary Figure 3 [file 41398_2026_4091_MOESM4_ESM.pdf]
