# Supplementary material for: The development of FEDUPP: feeding experimentation device users processing package to assess learning and cognitive flexibility
Source: Transl Psychiatry. 2026 May 16;16:348. doi: 10.1038/s41398-026-04091-6 (PMC13346605; doi:10.1038/s41398-026-04091-6)

Meal Metrics

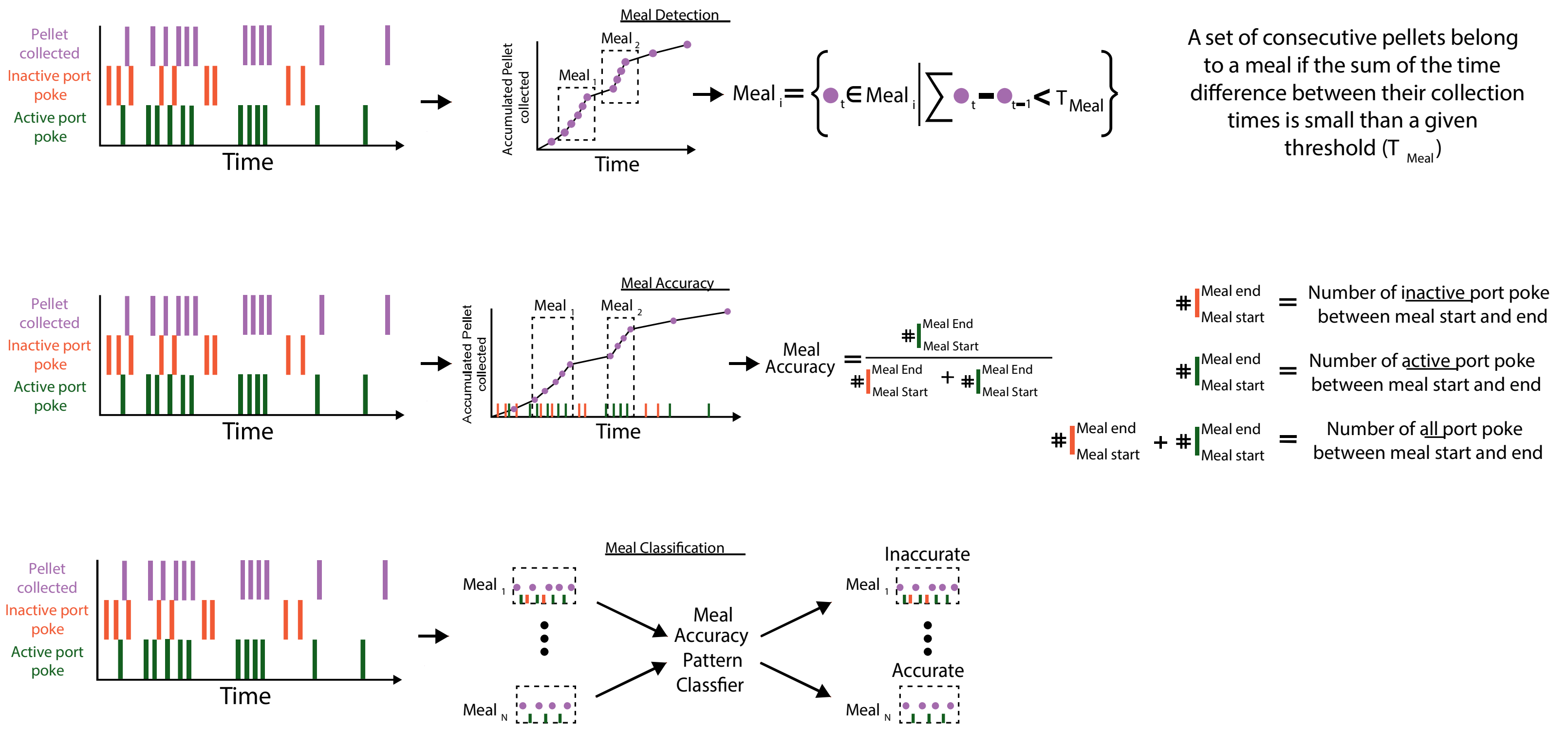

Supplement: Supplementary file 5 — Supplementary Figure 4 [file 41398_2026_4091_MOESM5_ESM.pdf]
