# Supplementary material for: The development of FEDUPP: feeding experimentation device users processing package to assess learning and cognitive flexibility
Source: Transl Psychiatry. 2026 May 16;16:348. doi: 10.1038/s41398-026-04091-6 (PMC13346605; doi:10.1038/s41398-026-04091-6)
Supplement: Supplementary file 6 — Supplementary Figure 5 [file 41398_2026_4091_MOESM6_ESM.pdf]

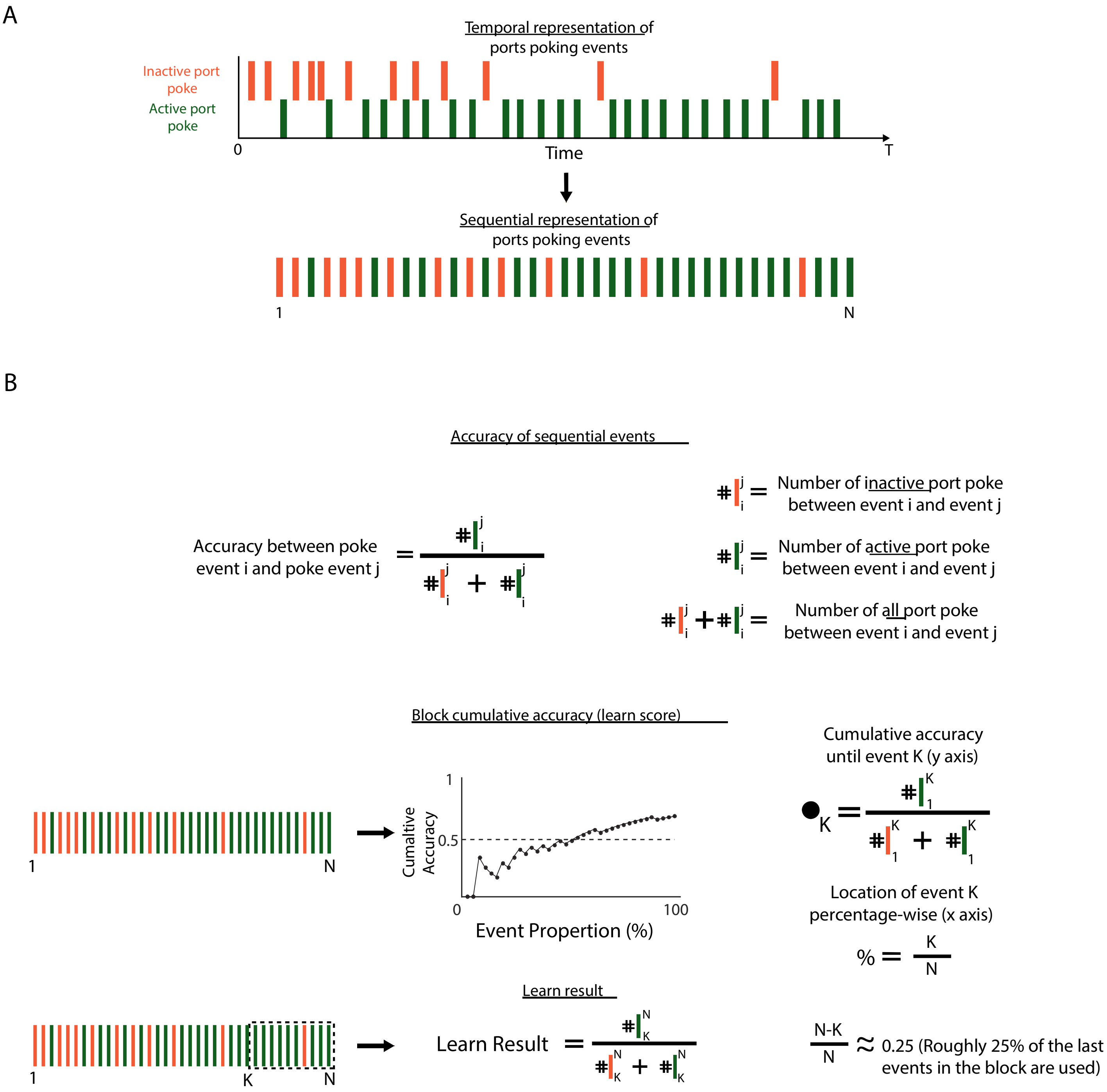
A
Temporal representation of
ports poking events
Inactive port poke
Active port poke
0
Time
T
Sequential representation of
ports poking events
1
N
B
Accuracy of sequential events
Accuracy between poke event i and poke event j
Number of inactive port poke between event i and event j
Number of active port poke between event i and event j
Number of all port poke between event i and event j
Block cumulative accuracy (learn score)
1
N
Cumaltive Accuracy
1
0.5
0
100
Event Propertion (%)
Cumulative accuracy until event K (y axis)
Location of event K percentage-wise (x axis)
Learn result
1
K
N
Learn Result
0.25 (Roughly 25% of the last events in the block are used)
